# Editorial Note: Photosynthesis

**DOI:** 10.1042/EBC-2016-0016C_EDN

**Published:** 2021-07-26

**Keywords:** membrane, photosynethesis, thylakoid

One of the responsibilities of academic publishers is to ensure that the publication record is maintained accurately. Therefore, Portland Press would like to inform the reader of the following.

We have been made aware of an article published in the *International Journal of Pharmaceutical Science Invention* which reproduces significant content from this article without citation or attribution. We have attempted to contact the Editorial Office of the journal, the Editor-in-Chief and members of the Editorial Board and the Publisher in order to resolve this issue but despite repeated attempts no response has been received.

We would like to inform the readers that we are aware that there is another version of the article available online and reassure you that this paper is the original and correct version of record.

